# Coronary allograft vasculopathy in post-heart transplant patients: pathogenesis and role of cardiac computed tomography in diagnosis—a comprehensive review

**DOI:** 10.1097/MS9.0000000000000942

**Published:** 2023-06-07

**Authors:** Vagisha Sharma, Siddharth Agarwal, Tanvi Grover, Anureet Malhotra, Harendra Kumar, Diksha M. Gowda, Yash Agarwal, Hitesh Bhatia, Samrat Babu Koirala, Satinder P. Singh

**Affiliations:** a Vardhman Mahavir Medical College and Safdarjung Hospital, New Delhi; b Kempegowda Institute of Medical Sciences, Bangalore; c College of Medicine and Sagore Dutta Hospital, Kolkata, West Bengal; d Pt. B.D.S. Post Graduate Institute of Medical Sciences Rohtak, India; e University of Oklahoma, Oklahoma City, OK; f University of Kansas Medical Centre, Kansas City, KS; g Dow University of Health Sciences, Karachi, Pakistan; h Nepalese Army Institute of Health Sciences, Kathmandu, Nepal; i Department of Radiology, The University of Alabama at Birmingham, AL

**Keywords:** cardiac imaging, computed tomography, coronary allograft vasculopathy, heart transplant

## Abstract

Coronary allograft vasculopathy, often known as cardiac allograft vasculopathy (CAV), is a substantial source of morbidity and mortality in people who have had heart transplants. Early detection and monitoring of CAV are crucial for improving outcomes in this population. Although cardiac computed tomography (CT) has emerged as a possible method for finding and evaluating CAV, invasive coronary angiography has long been thought of as the gold standard for recognizing CAV. This study focuses on the utility of cardiac CT for CAV diagnosis and treatment in the post-heart transplant population. It provides an overview of recent studies on the application of cardiac CT in CAV and highlights the advantages and disadvantages of this imaging modality. The potential application of cardiac CT for CAV risk assessment and care is also examined in the study. Overall, the data point to a potential role for cardiac CT in the detection and treatment of CAV in post-heart transplant patients. It enables evaluation of the whole coronary tree and low-radiation, high-resolution imaging of the coronary arteries. Hence, further study is required to determine how best to employ cardiac CT in treating CAV in this group.

## Introduction

Highlights

- Cardiac allograft vasculopathy (CAV) is a major cause of morbidity and mortality in heart transplant patients.
- Early detection is crucial for optimal patient outcomes
- Cardiac computed tomography (CT) accurately detects the presence and extent of CAV.
- Cardiac CT has a high negative predictive value for ruling out significant CAV.
- Cardiac CT has advantages over invasive angiography, including lower risk of complications, shorter procedure time, and lower cost.

Cardiac allograft vasculopathy (CAV) is a common complication in heart transplant patients, particularly in the first 5 years^1^. To timely diagnose Coronary Artery Vasculopathy in heart transplant recipients undergo a yearly invasive coronary angiography^2^. However, the International Society of Heart and Lung Transplant (ISHLT) has found certain limitations to this technique.

The pathogenesis of the condition involves immune-mediated coronary artery remodelling which includes diffuse concentric intimal hyperplasia—ultimately resulting in ischaemic graft failure and loss^3,4^. It is a type 4 hypersensitivity reaction, however non immunologic mechanisms similar to those in atherosclerotic heart disease also play a role in it^5^. Calcium content and necrotic core composition increase with time while fibrous components decrease^6^. It often occurs concurrently with coronary artery disease in transplanted vessels^7^. Allograft vasculopathy, however, can affect any region of the coronary artery, in contrast to atherosclerotic coronary artery disease, which develops proximally in the coronary vasculature and results in eccentric atherosclerotic constriction. Similar vasculopathy also takes place in other vascularized organ grafts.

The standard of diagnosis for CAV is invasive coronary angiography. However recent advances have led to coronary computed tomography angiography (CTA) as a potentially viable alternative to invasive screening—due to equivalent accuracy, cost-effectiveness and anatomical information yielded^8^. Coronary artery calcium obtained on cardiac CT is a marker of atherosclerotic heart disease in the general population; however, in heart transplant recipients, it can be. The absence of coronary artery calcification is correlated to a low prevalence of ISHLT. In the present paper, we try to demonstrate the ability of the noninvasive approach of dual-source CT coronary angiography to surpass invasive coronary angiography (ICA) in diagnosing stenotic CAV in heart transplant (HTX) recipients.

### Cardiac allograft vasculopathy

A common long-term complication following heart transplantation and a major contributor to late death is CAV. Although immunosuppressive medication has improved, the prevalence of CAV has hardly diminished and still affects up to 50% of transplant recipients within 10 years^9^. The prevalence of CAV is reported to be 7.8% in the first year following adult heart transplantation, 30% in the next 5 years, and 50% in the following 10 years, according to the ISHLT’s most recent registry data^10^. For cardiac transplant recipients who had their transplant more than 3 years ago, CAV is the third most common cause of death.

Over the past 20 years, there have been significant changes in the incidence of CAV, the available therapeutic choices, and the survival of transplant recipients with CAV. In patients transplanted between 1994–2002 and 2003–2011, the cumulative incidence of CAV at 8 years after transplant fell from 46 to 40%. Additionally, these patients’ survival has increased: the 5-year mortality rate among patients with CAV reported during the first 3 years post-transplant has fallen from 29% in the earlier age to 25% in the more recent era^11^.

### Pathogenesis and risk factors

**Figure 1:**
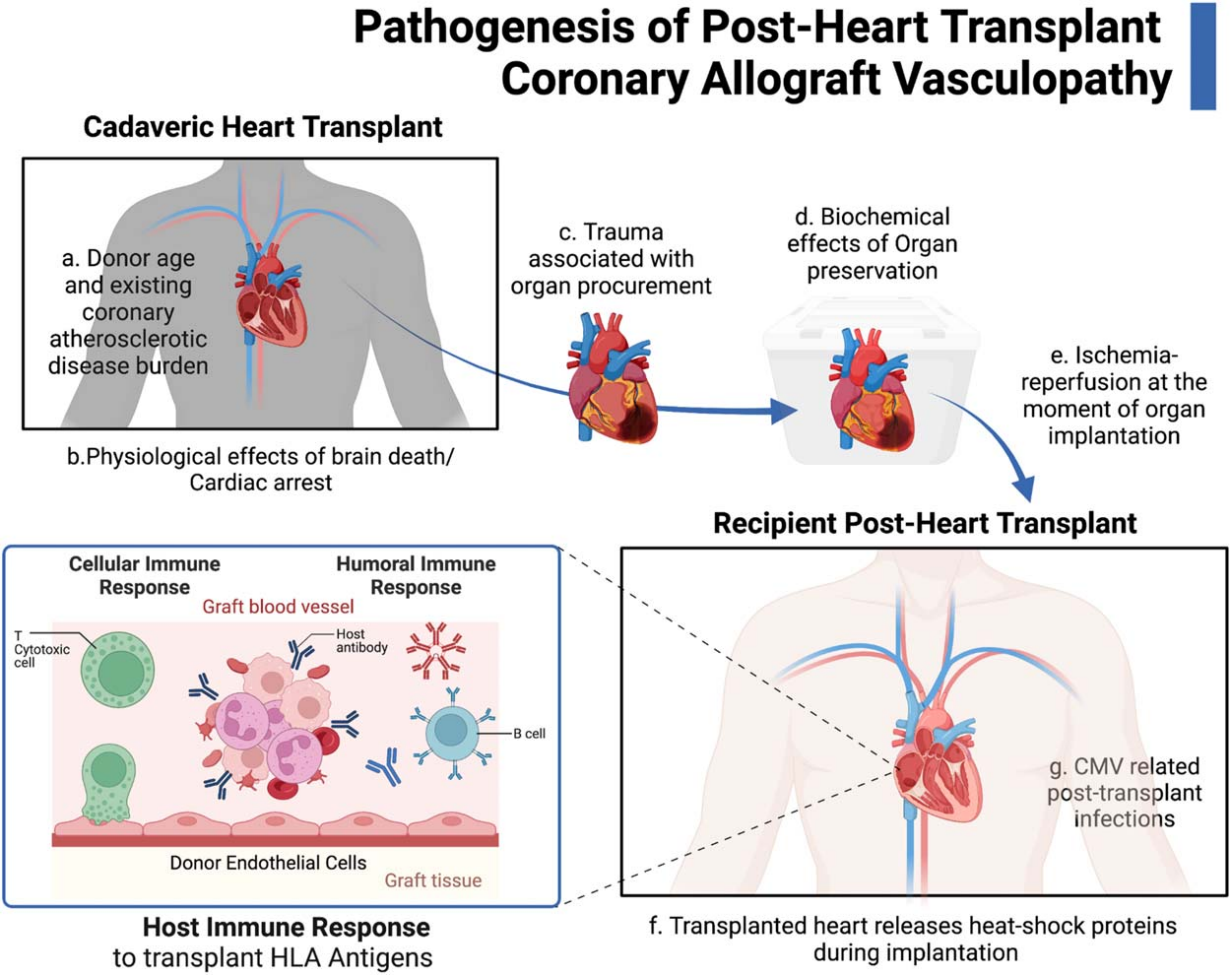
Pathogenesis of post-heart transplant coronary allograft vasculopathy.

Complex immunological and non-immunological mechanisms contribute to the aetiology of CAV. Inflammation and endothelial damage can be brought on by the donor’s cardiac arrest, organ donation, allograft ischaemia and reperfusion, and organ procurement. CAV is a result of a combination of innate and adaptive immunity. The donor’s heart releases heat-shock proteins and HLA antigens during implantation, which recipient antigen-presenting cells can process to activate T lymphocytes. The majority of the antigens that trigger the host immune system are produced by endothelial cells lining the arteries of allografts. In the allograft, donor-specific antibodies might develop against HLA or non-HLA antigens (vimentin, anti-cardiac myosin)^12^. Donor-specific antibodies, particularly those directed against MHC II antigens, have been linked to CAV and poor post-heart transplant outcomes. CAV may also be significantly influenced by non-HLA antibodies, many of which are antigens produced on endothelial cells. Antibodies to the angiotensin II type 1 receptor, anti-MHC class I chain-related A and B, adhesion and trafficking receptors, and others are examples of non-HLA antibodies. Proinflammatory cytokines are released as a result of immune system activation, which also causes endothelial damage, additional vascular inflammation, and myxoid changes in the intima of early lesions as well as fibrotic and hyalinized changes in advanced lesions. All of these factors contribute to the pathogenesis of CAV^13^ (Figure 1).

Traditional risk factors for coronary atherosclerosis and tissue injury at the time of organ procurement and implantation are examples of nonimmune-mediated pathways that most likely contribute to CAV. The physiologic changes associated with brain death, organ preservation, cardiac arrest of the donor’s heart before transplant, surgical trauma during organ procurement, and ischaemia-reperfusion at the moment of organ implantation are additional nonimmune variables that may contribute to CAV. CAV development has been linked to older donor ages. CMV-related post-transplant infections, in particular, are linked to the emergence of CAV^13^.

Hypertension, hyperlipidemia, and glucose intolerance are traditional risk factors for native coronary artery disease. These comorbid conditions are fairly common both before and after cardiac transplantation. According to data from the ISHLT registry, after 5 years of receiving a heart transplant, 38% of patients develop diabetes, 92% of patients experience hypertension, and 88% of patients have hyperlipidemia. Similar to how native coronary artery disease develops, CAV is likely influenced by hypertension, hyperlipidemia, and diabetes^13^.

### Prevention and treatment

**Figure 2:**
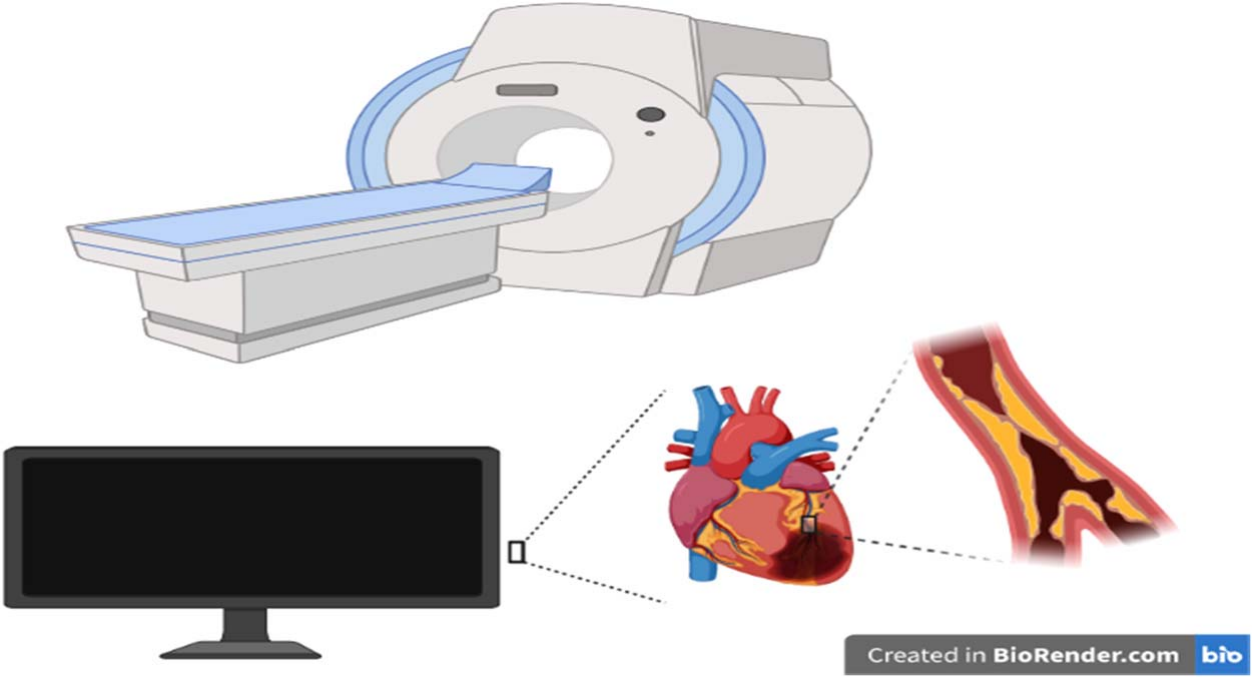
An illustration of the use of cardiac CT in coronary allograft vasculopathy. CT, computed tomography.

Early administration of the statins simvastatin and pravastatin after transplantation reduces the risk of CAV by reducing cholesterol levels and cardiac rejection with associated hemodynamic compromise. It also prevents natural killer cells from functioning and also may offer further protection by reducing its cytotoxicity. The development of CAV may also be slowed down by vitamins C and E by reducing oxidant stress^5^. Because of aspirin’s well-documented advantages in treating native coronary artery disease, it is frequently recommended daily^12^. The likelihood of infection, rejection and the emergence of CAV may be impacted by the use of induction treatment. Anti-thymocyte globulin, a T-cell-depleting drug, and IL-2 receptor antagonists, such as basiliximab, are frequently used medications. Anti-thymocyte globulin induction was observed to postpone the start of CAV but did not affect survival^14^. The use of routine induction therapy is still debatable because there has yet to be any conclusive evidence that it improves patient survival. However, it is anticipated that induction therapy will still be used for many patients in the initial postoperative period following heart transplantation given the elevated frequencies of allosensitized patients awaiting transplant. The majority of patients—about 33%—are allosensitized, according to the most recent ISHLT registry data^15^. A patient’s likelihood of antibody-mediated rejection following a heart transplant may be affected by increased allosensitization, which could then raise their risk of developing CAV (Figure 2).

Once CAV is identified, a proliferation signal inhibitor, such as sirolimus or everolimus, can be used to slow the course of the illness. Contrary to the combination of calcineurin inhibitors and purine antagonists, proliferation signal inhibitors and calcineurin inhibitors had a lower rate of CAV. Compared with cyclosporine and azathioprine, everolimus and cyclosporine exhibited decreased rates of CAV and CMV infection in de novo heart transplant recipients^13^. Everolimus inhibits smooth-muscle cell growth and T-cell proliferation, preventing CAV^16^. Percutaneous coronary procedures for localized disease can alleviate clinically severe CAV, but restenosis rates are substantial4. Adoption of drug-eluting stents has decreased in-stent restenosis rates in heart transplant patients to levels equivalent to those of the general population with coronary artery disease. Retransplantation is frequently the only practical choice; however, it poses issues with fair organ distribution. The use of implantable cardioverter-defibrillators may also be a successful method of reducing the risk of sudden cardiac death in patients with CAV^13^.

### Feasibility of dual-source computed tomography angiography (DSCTA) over other modalities for the detection of CAV

Heart transplant rejection does not have any clinically relevant biomarkers for detection. Therefore there are various invasive and noninvasive tools of assessment for detecting CAV. They are coronary angiography, stress echo, intravascular ultrasound, optical coherence tomography, positive electronic tomography and CT angiography (Table 1).

**Table 1:** Advantages and disadvantages of different modalities in diagnosing CAV

Modality	Advantages	Disadvantages
Coronary angiography	Gold standard for assessing coronary artery anatomy Ability to intervene and treat any identified blockages during the same procedure	Invasive procedure that carries risks of bleeding, infection, and radiation exposure Limited visualization of vessel wall and plaque composition
Stress echo	Noninvasive and does not involve radiation exposure Can assess both functional and structural aspects of the heart Ability to perform dynamic imaging during stress testing	Limited ability to visualize coronary artery anatomy and plaque composition Not suitable for patients with certain comorbidities, such as severe lung disease
Intravascular ultrasound	Provides high-resolution imaging of the vessel wall and plaque composition Ability to assess the severity of a blockage and guide treatment Can be used during coronary angiography procedure	Invasive procedure that carries risks of bleeding and infection Does not provide information on the functional status of the heart
Optical coherence tomography	Provides high-resolution imaging of the vessel wall and plaque composition Ability to assess the severity of a blockage and guide treatment Can be used during coronary angiography procedure	Invasive procedure that carries risks of bleeding and infection Limited visualization depth and field of view
Positron emission tomography	Can provide information on myocardial perfusion and metabolism Noninvasive and does not involve radiation exposure	Limited spatial resolution and field of view Not widely available and can be expensive
Dual scan CT	Noninvasive and does not involve radiation exposure High accuracy in detecting coronary artery disease Can assess both functional and structural aspects of the heart	Limited ability to assess plaque composition Risk of false positive results

CAV, cardiac allograft vasculopathy; CT, computed tomography.

Dobutamine stress echo has 70–80% sensitivity for detecting angiographical significant CAV. Intravascular ultrasound even though invasive detects silent angiographic lesions and visualizes the vessel wall but fails to detect smaller vessels due to the larger size of the catheter for narrower vessels. Optical coherence tomography help in characterising CAV, and defining the composition of the plaque. PET quantifies myocardial perfusion to detect microvascular disease.

ICA, even though ISHLT recommended modality and the gold standard to assess the lumen patency for detection and quantification of CAV. It is associated with a risk of complications such as bleeding, infections, stroke, myocardial infarction allergy to the contrast dye, coronary artery dissection and higher cost and it misses out some lesions^17^.

The development of Dual Scan CT has brought a new outlook for cardiovascular imaging. It offers to obtain visual unenhanced images and virtual monochromatic images at different energy levels. DSCTA evaluates CAV with good image quality and high diagnostic accuracy and also rules out the pathology due to a high negative predictive value. Schepis and colleagues report sensitivity, specificity, and positive and negative predictive values of 85%, 84%, 76%, and 91%, respectively, for the detection of CAV examined by DSCTA in 30 patients after HTX compared with ICA and 1-vessel IVUS^18^.

According to Salavati and colleagues, a patient-based meta-analysis to diagnose and assess CHD of 2303 patients using DSCTA shows pooled sensitivity and specificity of 99% and 89%. The study also shows a robust high heart rate^19^. Therefore beta-blockers were used before DSCTA to reduce the heart rate and improve the image quality^20^. Nous and colleagues study used intravenous beta-blockers in HTx patients and the heart rate was reduced by 15%^21^. Von Ziegler and colleagues study presents with clinical feasibility of DSCTA to detect significant coronary artery stenosis after heart transplantation due to CAV. The study had segment-based analysis with sensitivity, specificity and diagnostic accuracy of 100%, 98.9%, and 98.9%, respectively, and also had patient-based analysis with sensitivity, specificity and diagnostic accuracy of 100%, 86%, and 93%, respectively. The study showed a high negative predictive value of 100%, which suggests DSCTA is a useful tool to rule out stenosis in heart transplant recipients and makes ICA unnecessary to be done annually Anne this stops further investigations in patients^22^. In another study, Anne and colleagues also showed high negative predictive value using Coronary CT angiography which helps rule out significant stenosis for intervention^23^. This was supported by Rohnean et al study, 5 years after which he followed up 65 transplant recipients by conventional computed tomography angiography. The findings suggested conventional computed tomography angiography is the safest noninvasive tool for monitoring heart transplant recipients. Moreover, the time required to develop stenosis was greater than 3 years, thus a 2-year follow-up interval, when there’s a normal baseline, would be safe. This removes the need for conventional coronary angiography^24^.

Cardiac CT is a noninvasive diagnostic imaging technique that uses X-rays to provide detailed images of the heart and its components. Cardiovascular CT may be useful in identifying structural abnormalities or changes in blood flow that may be related to CAV^21,24^.

The existence of coronary artery stenosis or narrowing may be seen on cardiac CT in those with CAV. This occurs when plaque accumulates inside the walls of the coronary arteries, reducing the amount of blood flow to the heart muscle. Symptoms may include chest pain or discomfort, shortness of breath, and tiredness^11,23^.

Evidence of myocardial ischaemia, or decreased blood flow to the heart muscle, may also be shown on cardiac CT in those with CAV. This may be seen as areas of decreased contrast enhancement or perfusion inside the myocardium, which might indicate areas of ischaemia or infarction^22^. Evidence of calcified or non-calcified plaque, which may further restrict the coronary arteries and increase the risk of myocardial infarction or other cardiovascular events, may also be seen on cardiac CT in persons with CAV^23^.

Overall, cardiac CT may be a useful diagnostic method for identifying and diagnosing structural and functional CAV abnormalities. Cardiac CT, by providing accurate images of the heart and its blood vessels, may help clinicians better understand the underlying pathophysiology of the disease and provide specific therapeutic choices to improve patient outcomes^14^.

### Cardiac computed tomography in paediatric population

CT scanning is a useful imaging modality for paediatric patients for a wide range of diagnostic objectives^25^. CT uses X-rays and computer processing to generate comprehensive images of the body, which may be particularly useful in children who may be unable to remain still for long periods of time during traditional imaging procedures^26^.

The assessment of head trauma is a common purpose for CT in paediatrics. CT can quickly and accurately detect cerebral haemorrhage or other traumatic injuries, which may be fatal if not recognized and treated promptly. CT may also be utilized in paediatric patients to investigate various types of head and neck disorders, such as brain tumours, hydrocephalus, and sinusitis^27^.

In paediatric patients, CT scans of the chest and abdomen may also be employed. CT scans of the chest may be performed to look for signs of pulmonary embolism, pneumonia, or congenital abnormalities. CT scans of the abdomen may be performed to look for signs of appendicitis, intestinal obstruction, or other abdominal diseases^25,26^.

While CT is an important diagnostic tool in paediatrics, there are certain risks associated with ionizing radiation exposure, particularly in young children, who are more vulnerable to the effects of radiation. Radiologists and other healthcare professionals should follow established guidelines for imaging paediatric patients, including the use of low-dose protocols and alternative imaging modalities such as ultrasound or MRI when appropriate, to minimize radiation exposure^27,28^.

CT may be a useful diagnostic technique in paediatrics for a number of reasons, particularly when prompt and accurate diagnosis is critical for patient outcomes. However, it is critical for healthcare practitioners to weigh the benefits and risks of CT imaging in each case and to use appropriate methods and practices to decrease radiation exposure^29^.

### Limitation of DSCTA

DSCTA is an imaging method that may be used to evaluate CAV in heart transplant patients^30^. However, it is not yet a regular procedure and has various limitations. The primary limitation of DSCTA is associated with image quality, which is heavily influenced by characteristics such as a fast heart rate and low reactivity to beta-blockers in heart transplant patients. Furthermore, using cardiac CT to diagnose CAV exposes the patient to significant radiation, which is especially concerning for young individuals^2^. As a result, surgeons must weigh the potential benefits of surgery against the risks of radiation exposure^31,32^.

Another disadvantage of DSCTA is its inability to see the heart’s microvasculature, which may be compromised in heart transplant patients with CAV. Other imaging modalities, such as cardiac MRI, may be better suited for studying the heart’s microvasculature^33^.

Moreover, DSCTA requires the injection of iodinated contrast chemicals, which may cause unpleasant reactions in certain individuals. Individuals with a history of contrast allergy or renal impairment should avoid using contrast agents^33^.

DSCTA is a promising tool for identifying CAV in heart transplant patients, despite severe limitations related to image quality, radiation exposure, and the inability to visualize the heart’s microvasculature. When considering its use in the evaluation of cardiac transplant patients with suspected CAV, physicians must weigh the possible benefits of the therapy against its limitations.

### Cardiac MRI

Coronary allograft vasculopathy (CAV) is a frequent complication after heart transplantation that affects up to 50% of patients within 10 years of surgery^34^. It is distinguished by extensive, concentric, and rapidly developing intimal hyperplasia of the epicardial coronary arteries, which leads to myocardial ischaemia, infarction, and cardiac failure. Because CAV is difficult to detect in its early stages and is usually asymptomatic, frequent screening is essential for rapid diagnosis and treatment. Cardiac MRI is a noninvasive imaging technology that has emerged as a valuable tool for detecting CAV in young heart transplant recipients ^34,35^. Cardiac MRI is a versatile imaging technique that uses magnetic fields and radio waves to provide detailed images of the heart and blood vessels. It may provide information on cardiac function, design, and perfusion, making it an appropriate method for evaluating heart transplant candidates. Several studies have been conducted in recent years to investigate the role of cardiac MRI in the diagnosis of CAV in paediatric heart transplant patients^36,37^. One of the key advantages of cardiac MRI is its ability to provide accurate and comprehensive information on the structure of the coronary arteries. MRI may detect early changes in the coronary arteries, such as intimal thickening and luminal constriction, both of which are symptoms of CAV^38^. Several studies have shown that cardiac MRI is very sensitive and specific for detecting CAV in paediatric heart transplant patients^39^. Aside from providing information on coronary geometry, cardiac MRI may also evaluate myocardial perfusion and function, which can help in the diagnosis and treatment of CAV. Cardiac MRI may detect myocardial ischaemia, a common sign of CAV, and provide information on the severity and extent of the ischaemia. It may also look at ventricular function, which is important in understanding how CAV affects cardiac function. Several studies have shown that cardiac MRI may detect myocardial ischaemia and dysfunction in CAV-adolescent heart transplant patients^38,40^. Another advantage of cardiac MRI is that it is noninvasive. In contrast to invasive imaging techniques such as coronary angiography, cardiac MRI does not require the use of contrast agents or radiation, making it a safer and more patient-friendly approach to monitoring CAV in paediatric heart transplant patients. Furthermore, cardiac MRI may be performed serially without the risk of cumulative radiation exposure, which is important for monitoring disease progression and therapeutic response in young heart transplant patients with CAV^39,40^. Cardiac MRI is an effective tool for detecting CAV in young heart transplant recipients. Because of its ability to provide detailed information on coronary geometry, myocardial perfusion, and function, it is an ideal modality for evaluating CAV in this patient population. Furthermore, since it is noninvasive and does not expose patients to radiation, it is a safe and patient-friendly option for serial screening and monitoring of CAV in juvenile heart transplant patients. With advancements in technology and imaging processes, cardiac MRI is expected to become an increasingly important tool in the detection and treatment of CAV in paediatric heart transplant patients^31,39^.

## Conclusion

In conclusion, coronary allograft vasculopathy in post-heart transplant patients may be identified and monitored using cardiac CT, showing encouraging outcomes. CT offers a noninvasive option for routine monitoring and risk assessment, though traditional invasive angiography continues to be the gold standard. It now provides greater resolution and quicker acquisition times as a result of technical developments, making it a more practical choice for frequent clinical usage. However, additional research is needed to thoroughly investigate its advantages compared to current methods and potential impact on patient outcomes. Nonetheless, cardiac CT is a useful technique that should be taken into account as part of an all-encompassing monitoring strategy since it may help with the care of post-transplant patients with coronary allograft vasculopathy.

## Ethics approval and consent to participate

Not applicable.

## Source of funding

NA.

## Author contribution

All the authors contributed equally to the research.

## Conflicts of interest disclosure

The authors declare that there is no conflict of interest.

## Research registration unique identifying number (UIN)

NA.

## Guarantor

Samrat Babu Koirala.

## Data availability statement

NA.

## Provenance and peer review

Not commissioned, externally peer-reviewed.
